# Draft genome sequences of *Arthrobacter* sp. AZCC_0090 and *Mycobacterium* sp. AZCC_0083 isolated from oligotrophic subsurface forest soil in the Santa Catalina mountains of Southern Arizona

**DOI:** 10.1128/mra.01089-23

**Published:** 2024-02-20

**Authors:** Melanie R. Kridler, Isabella A. Viney, Joy M. Custer, Bradley Schlottman, Ryan Bartelme, Paul Carini

**Affiliations:** 1 Department of Environmental Science, University of Arizona, Tucson, Arizona, USA; 2 BIO5 Institute, University of Arizona, Tucson, Arizona, USA; California State University San Marcos, San Marcos, California, USA

**Keywords:** environmental microbiology, soil microbiology, genomics, microbial ecology, genome sequences

## Abstract

Here, we present the genomes of two soil actinobacteria: *Arthrobacter* sp. strain AZCC_0090 and *Mycobacterium* sp. strain AZCC_0083, isolated from oligotrophic subsurface soils in Southern Arizona, USA.

## ANNOUNCEMENT

Deep soil horizons contain microbes crucial for subsurface biogeochemistry and the global carbon cycle. While microbial communities have been extensively studied in surface horizons, we know far less about the microbial communities residing in deeper soil horizons despite their important roles in the mineralization of nutrients necessary for plant life (1–3).

Arizona Culture Collection (AZCC) cultures *Arthrobacter* sp. AZCC_0090 and *Mycobacterium* sp. AZCC_0083 were isolated from 50 cm subsurface soil at Oracle Ridge in Tucson, AZ, USA as described elsewhere (3) (Table 1). The cultures were propagated from isolated colonies on yeast mannitol medium (YM) consisting of (L^−1^) 1.0 g yeast extract, 10.0 g mannitol, 0.5 g K_2_HPO_4_, 0.2 g MgSO_4_, and 0.1 g NaCl. Solid YM medium was prepared by including 15 g L^−1^ Noble agar. The cultures were incubated at 20°C. Broth cultures were shaken at 180 RPM. Growth appeared within 4 days.

**TABLE 1:** Isolation information, genome accessions, and summary statistics

Strain	*Arthrobacter* sp. AZCC_0090	*Mycobacterium* sp. AZCC_0083
Isolation site and soil depth	Oracle Ridge 50 cm	Oracle Ridge 50 cm
Isolation site coordinates	32°27′1″ N 110°44′29″ W	32°27′1″ N 110°44′29″ W
GenBank accession	JACHEE000000000	JACHGP000000000
Genome accession	PRJNA632064	PRJNA632065
SRA ID	SRX8890241	SRX8890242
Biosample accession	SAMN14915971	SAMN14915993
Number of raw reads generated	16,660,990	18,463,710
Number of raw bp generated	2,515,809,490	2,788,020,210
Number of reads after filtering	16,539,064	18,410,218
Number of bp after filtering	2,479,728,727	2,759,045,573
*N*50	315,689	428,479
Scaffolds	34	40
Genome size (bp)	4,921,994	8,615,464
Percent GC	63.32	66.11
Average coverage (fold)	305	174
Contigs	41	47
Extrachromosomal elements	0	0
Protein-coding genes	4,653	8,566
5S rRNA	1	1
16S rRNA	1	3
23S rRNA	1	1
tRNAs	52	82
Putative CRISPR count	2	0
Putative biosynthetic gene clusters	0	40
Predicted viruses^*a*^	0	1
Completeness^*b*^	99.97	100
Contamination^*b*^	0.37	1.41

^*a*^ Predictions from geNomad (4).

^*b*^ Predictions from CheckM2 (5).

Genomic DNA (gDNA) was isolated from a broth culture originating from a single colony using the Joint Genome Institute CTAB protocol for bacterial DNA isolation (6). For AZCC_0083, 100 ng of gDNA was sheared to 511 bp using the Covaris LE220 and size selected with SPRI using TotalPure NGS beads (Omega Bio-tek) to enrich for 200–500 bp fragments. For AZCC_0090, 200 ng of gDNA was sheared to 461 bp. After size selection with double-SPRI to enrich for 200–500 bp fragments, DNA fragments were end-repaired, A-tailed, and ligated with Illumina-compatible sequencing adapters containing a unique molecular index barcode for each sample library using the KAPA-HyperPrep kit (KAPA biosystems).

The libraries were quantified using KAPA Biosystems’ next-generation sequencing library qPCR kit and run on a Roche LightCycler 480 real-time PCR instrument. Genomes were generated from Illumina 2 × 151 bp libraries using the Illumina NovaSeq XP V1 reagent kits and the S4 flowcell. Illumina sequences were quality filtered using BBTools: BBDuk (version 38.75) and BBMap and assembled with SPAdes (version v3.13.0; –phred-offset 33 –cov-cutoff auto -t 16 -m 64 –careful -k 25,55,95) (7, 8). Contigs < 1 kb were discarded (BBTools reformat.sh: minlength = 1,000 ow = t). Annotations were made using the prokaryotic genome annotation pipeline (9) and are available in IMG/MER (10). Both genomes are nearly complete, with minimal contamination via CheckM2 (5) (Table 1). Strain AZCC_0083 codes for 40 predicted biosynthetic gene clusters, and AZCC_0090 codes for two putative CRISPR genes (Table 1).

## Data Availability

The draft genome sequences of *Arthrobacter sp.* AZCC_0090 and *Mycobacterium sp*. AZCC_0083 have been deposited at NCBI. Their accessions are listed in Table 1.
